# Perforated Acute Appendicitis Presenting as Pneumoperitoneum in a Preterm Neonate: A Case Report and Literature Review

**DOI:** 10.1155/cris/5530326

**Published:** 2026-01-24

**Authors:** Ghadia A. AlAbidi, Mohammed S. Mallick, Ali Al-Ameer, Abdulrahman Alwahbi

**Affiliations:** ^1^ Department of Pediatric Surgery, King Saud Medical City, Riyadh, Saudi Arabia, ksmc.med.sa; ^2^ Department of Pediatric Surgery, King Fahad Medical City, Riyadh, Saudi Arabia, kfmc.med.sa; ^3^ Department of Pediatrics, King Saud Medical City, Riyadh, Saudi Arabia, ksmc.med.sa

**Keywords:** neonatal appendicitis, perforated appendicitis, pneumoperitoneum, premature neonate, surgical exploration

## Abstract

Acute appendicitis is rare in the neonatal population, and delayed diagnosis is associated with increased morbidity and mortality. Pneumoperitoneum is infrequently identified on plain radiography in cases of neonatal appendicitis, which can result in delays in diagnosis and intervention. In this case, we report a 15‐day‐old preterm female neonate who presented with irritability and abdominal distention. A plain abdominal radiograph demonstrated significant pneumoperitoneum, and surgical exploration revealed perforated appendicitis.

## 1. Introduction

Appendiceal perforation is rare in the neonatal period compared to its incidence in older children [1]. The appendix is a narrow, blind‐ended tubular structure projecting from the cecum, and in neonates, its length ranges from approximately 1.49 to 8.14 cm [1]. The relatively wider appendiceal base in neonates reduces the likelihood of luminal obstruction and the risk of acute appendicitis [1]. In this case report, we present a preterm neonate who developed pneumoperitoneum and was initially suspected to have necrotizing enterocolitis but was ultimately diagnosed with perforated appendicitis following laparotomy. This represents a rare pathology with an atypical clinical presentation.

## 2. Case Presentation

**Figure 2:**
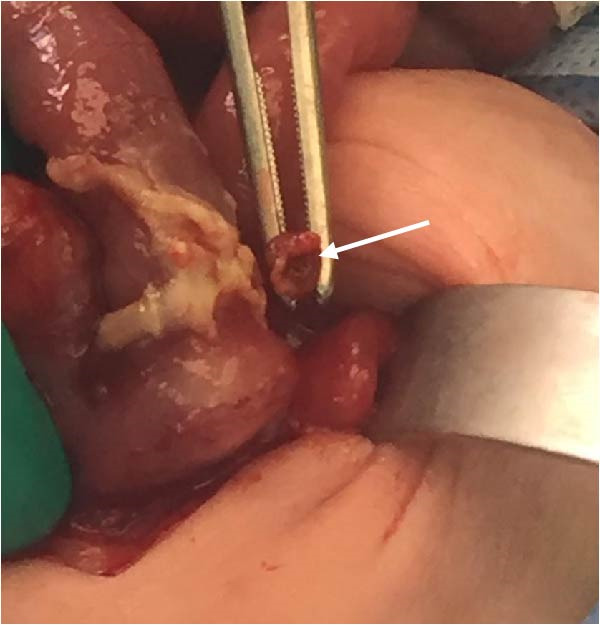
Perforated appendicitis.

**Figure 3:**
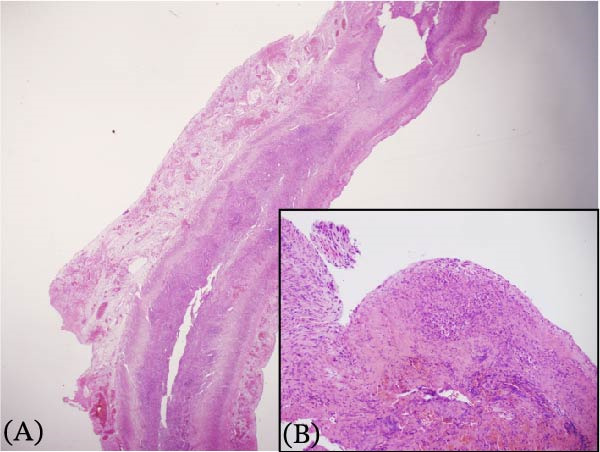
Histology of the appendix with hematoxylin and eosin staining showing evidence of appendicitis and periappendicitis. (A) A low‐power view showing the loss of normal architecture, infiltration of inflammatory cells, and congestion in subserosal blood vessels. (B) A high‐power view showing infiltrating cells, primarily neutrophils, along with fibrosis and fibrin deposition.

A preterm female neonate was born at 27 weeks’ gestation via spontaneous vaginal delivery to a 30‐year‐old mother (G3P2 + 0) with no significant medical history. The infant’s Apgar scores at 1 and 5 min were 7 and 8, respectively, and her birth weight was 1000 g. She was admitted to the Neonatal Intensive Care Unit for prematurity and respiratory failure and was started on intravenous ampicillin and gentamicin by the attending neonatologist. At 15 days of life (corrected gestational age: 29 weeks), the neonate developed episodes of irregular breathing associated with recurrent bradycardia, oxygen desaturation, and progressive abdominal distention. She was kept nil per os and was intubated for respiratory failure. Plain abdominal radiography revealed pneumoperitoneum (Figure 1). Emergency laparotomy was performed. Intraoperative examination revealed a hyperemic appendix with perforation at the tip (Figure 2). The small and large intestines appeared normal, and the remaining abdominal viscera were unremarkable. An appendectomy was performed, and the specimen was submitted for histopathological analysis, which confirmed acute appendicitis with periappendicitis (Figure 3). The infant recovered without complications and was discharged in stable condition.

Figure 1Abdominal radiographic images showing pneumoperitoneum (arrows) in the (A) supine position and in the (B) supine cross‐table lateral view.

**Figure:**
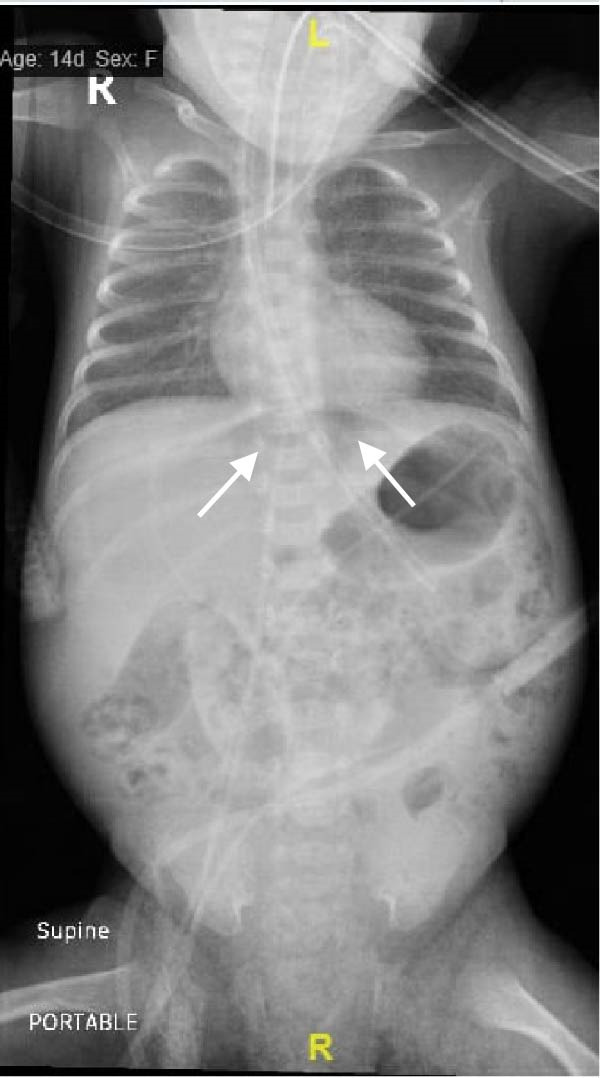
(A)

**Figure:**
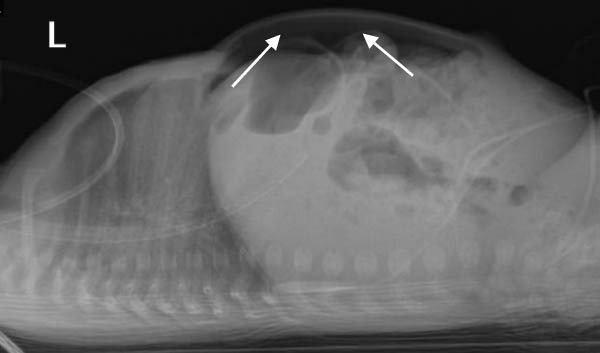
(B)

## 3. Discussion

In neonates, acute appendicitis is a rare cause of abdominal sepsis and is often not considered in the initial differential diagnosis. According to Stiefel et al., [2] the incidence of neonatal appendicitis is approximately 0.04%–0.20%. El‐Gohary and Al jubouri [3] observed that up to 25%–50% of neonatal appendicitis cases occurred in premature infants. Anatomical and physiological factors may contribute to its low incidence. In neonates, the appendix often has a funnel‐shaped base with a wide opening into the cecum, in contrast to the fingerlike form observed in older children [1]. Additionally, the likelihood of intraluminal obstruction is reduced due to factors such as a liquid diet, a recumbent position of the appendix, and the infrequent occurrence of intestinal and respiratory infections [4]. Diagnosing neonatal appendicitis is challenging due to vague and nonspecific clinical manifestations. These may include feeding intolerance, abdominal distension, irritability, and respiratory distress, which are often mistaken for other neonatal conditions like necrotizing enterocolitis. A low index of suspicion further contributes to delayed diagnosis. These delays can lead to high morbidity and mortality, as abdominal sepsis may progress to septic shock [5] or tension pneumoperitoneum secondary to perforated, gangrenous appendicitis [6], which can cause progressive end organ dysfunction and potentially fatal outcomes [7]. The high risk of perforation is also attributed to a thin‐walled appendix, an indistensible cecum, and a small, underdeveloped omentum [8].

Abdominal radiography is usually the first imaging tool in suspected case of neonatal abdominal sepsis; however, ultrasonography and CT are also used [9]. Pneumoperitoneum is rarely identified on plain radiographs—reported in approximately 8% of perforated appendicitis cases [10]. This makes the radiograph finding in our patient both unique and diagnostically valuable (Figure 1). Although the etiology of neonatal appendicitis is often unclear, predisposing factors include poor immunity, intestinal ischemia, and mechanical obstruction caused by conditions such as Hirschsprung’s disease, meconium plug syndrome, or cystic fibrosis [11]. Some authors suggested that neonatal appendicitis may represent a localized form of necrotizing enterocolitis [12]. Therefore, histological examination of the appendix—and, where indicated, the colon and rectum—may help rule out associated pathologies such as Hirschsprung’s disease [11]. Surgical intervention remains the mainstay of treatment. In the absence of underlying conditions such as Hirschsprung’s disease, appendectomy with peritoneal lavage remains the treatment of choice [13].

In conclusion, neonatal appendicitis is a rare condition with a nonspecific clinical presentation, making timely diagnosis particularly challenging. Delayed diagnosis and management are associated with significant morbidity and mortality. To improve outcomes, neonatologists and pediatric surgeons must be vigilant and consider acute appendicitis in the differential diagnosis of neonates presenting with abdominal symptoms.

## Funding

No funding or grants were received for this study.

## Disclosure

This case report was presented as poster presentation at WOFAPS 2022.

## Consent

All patient information and materials were carefully managed to prevent any unintended disclosure of personal data. All identifiable details were removed from the case report figures to ensure patient confidentiality.

## Conflicts of Interest

The authors declare no conflicts of interest.

## Data Availability

Data sharing is not applicable to this article as no datasets were generated or analyzed during the current study.
